# Communities and employers show a high level of preparedness in supporting working mothers to combine breastfeeding with work in rural Kenya

**DOI:** 10.1111/mcn.13180

**Published:** 2021-04-15

**Authors:** Taddese Alemu Zerfu, Paula Griffiths, Teresia Macharia, Eva W. Kamande, Esther Anono, Laura Kiige, Peter Muriuki Gatheru, Susan Jobando, Grainne Moloney, Elizabeth Wambui Kimani‐Murage

**Affiliations:** ^1^ Global Academy of Agriculture and Food Security University of Edinburgh (UoE) UK; ^2^ African Population and Health Research Center Nairobi Kenya; ^3^ International Livestock Research Institute (ILRI) Kenya Nairobi Kenya; ^4^ Loughborough University UK; ^5^ UNICEF Nairobi Kenya

**Keywords:** breastfeeding, combine, employed mother, work

## Abstract

Community Readiness Model (CRM) through pragmatic mixed methods design, combining quantitative CRM survey with qualitative data, was used to assess the level of preparedness and readiness among local leaders, employers and community members in supporting working mothers to combine breastfeeding with work. The study was conducted in one of the tea state farms in Kericho County of Kenya. A total of 17 purposively selected men (fathers), lactating mothers, peer educators, health professionals (doctors, nurses and nutritionists), tea plantation managers and grandmothers were interviewed. The CRM that has six different dimensions was applied to determine the stage of readiness to support working mothers to combine breastfeeding with work. Community Readiness Score (CRS) was calculated descriptively as mean ± standard deviation (SD). Thematic analysis using NVIVO software was used to analyse qualitative data. We found that the mean (±SD) CRS was 7.3 (1.9), which corresponded to the third highest level of the nine stages or the ‘stabilization’ stage of community readiness. Dimensionally, the mean CRS was the highest (8.3 ± 1.9) for leadership followed by community efforts (7.5 ± 2.1), whereas the lowest CRS was observed for knowledge of efforts (6.6 ± 2.3) and availability of resources (6.6 ± 1.9). In conclusion, high level of readiness to support working women to combine work with breastfeeding with suboptimal knowledge of efforts and availability of resources was observed in the area. Future interventions should focus on enabling the community to feel more comfortable and creating detailed and refined knowledge on combining breastfeeding with work.

**Key messages**

- The mean readiness score to supporting working mothers to combine their work with breastfeeding was 7.3, which corresponds to the third highest level of the nine community readiness stages suggesting high level of preparedness and readiness for support for breastfeeding in mothers returning to work in the agricultural plantation.
- Programmes and policies, in place, addressing support for combining breastfeeding with work are supported by local decision makers, company managers/supervisors and other community members.
- However, knowledge of efforts and availability of resources remained suboptimal.
- More efforts are still needed so that community members feel comfortable using services, and local data are regularly obtained.
- Actions towards detailed and sophisticated knowledge about prevalence, causes and consequences of unsupported working mothers to combine their work with breastfeeding are needed.

## 1 INTRODUCTION

Good nutrition promotes human biological development essential for survival across the entire human lifespan (Victora & Barros, 2000). Breastfeeding during early life, in particular, is crucial for lifelong health benefits, optimal psychosocial development and stimulating child–mother bonding (Moore et al., 2016).

Breastfeeding is a critical aspect of caring for infants and young children, as breast milk remains the single most important natural first food for babies (World Health Organization, 2018). It provides all energy and nutrients requirements for infants, promotes sensory and cognitive development and protects them against infectious and chronic diseases (Salone et al., 2013; World Health Organization, 2018).

Breastfeeding also contributes to the health and well‐being of mothers, helping to space children, reducing the risk of cancer (ovarian and breast), increasing family and national resources and acting as a secure way of feeding and is safe for the environment (Furman & Schanler, 2017). Exclusive breastfeeding in particular reduces infant mortality related to common childhood illnesses such as diarrhoea or pneumonia and helps for a quicker recovery during illness/infection (Quigley et al., 2007).

On the other hand, irrespective of the high awareness levels, favourable attitudes and continued advocacy for breastfeeding, breastfeeding rates in general and exclusive breastfeeding practices, in particular, remain quite low among employed mothers in many parts of the world (Bonet et al., 2013; Dodgson et al., 2004; Lakati et al., 2002b). In Kenya, for example, only 13.3% and 5% of infants of employed mothers were fed with exclusive breastfeeding at 3 and 6 months, respectively. Breast milk insufficiency and return to work are the main reasons for early cessation of exclusive breastfeeding (Lakati et al., 2002b).

Employed mothers often face difficulties to maintain exclusive breastfeeding when returning to the workplace (Lakati et al., 2002b). Accumulating evidence shows that postpartum return to work is associated with shorter breastfeeding duration (Lewallen et al., 2006; Roe et al., 1999) and lower breastfeeding intensity (Roe et al., 1999).

Presence and use of social support groups, less mother–infant separations, supportive work environments and facilities and child care options have been identified as critical factors facilitating the combining of work with breastfeeding globally (Johnston & Esposito, 2007; Lakati et al., 2002a; Majee et al., 2016; States & Committee, 2010). Particularly, the presence of lactation breaks in the workplace are among the useful ways to improve the health and productivity of working women and their children (Barbosa, 2015; States & Committee, 2010).

However, in many parts of the world, women feel largely unsupported by managers and their organizations, affecting the exclusive and continued breastfeeding rates among working mothers (Dodgson et al., 2004; Jantzer et al., 2018; A. M. Johnson et al., 2015; K. M. Johnson & Salpini, 2017; Majee et al., 2016; Weber et al., 2011). Therefore, there is an urgent need for a community‐ and workplace‐based multifaceted programme promoting engaging employers into principles that focus on the support for breastfeeding mothers after they leave the hospital (Kavle et al., 2019). In Kenya, community support for breastfeeding is being implemented through the Governments Baby Friendly Community Initiative (BFCI), and this provides a viable model for scaling to include workplace support for breastfeeding because it has at the heart of its approach a focus on working with communities to support optimal infant feeding practices.

Before beginning to develop a community‐based programme that includes workplaces as part of the model, there is a need to understand the stage of readiness of key stakeholders to establish whether the needed support to develop such an intervention exists among stakeholders such as employers and local communities. Evidence on the level of preparedness and readiness of communities and decision makers to support working mothers to combine work with breastfeeding is very lacking in low‐ and middle‐income country contexts including Kenya.

Initially designed to assess perceptions of readiness for interventions targeted at reducing consumption of alcohol, tobacco or other drugs, the Community Readiness Model (CRM) often serves as a theoretical framework for understanding and improving community readiness of a new intervention and aids design of effective and tailored interventions (Engstrom & Jason, 2002; Plested et al., 2006). The term readiness is defined as ‘the degree to which a community is prepared to take action on an issue’ while also referring to the observable and psychological factors that affect the ability of a community to change (Beebe et al., 2001). Therefore, the aim of this study is to explore the level of preparedness of the local community, employer and other stakeholders to support employed mothers to combine breastfeeding with work in an agricultural setting in rural Kenya.

## 2 METHODS

### 2.1 Study design

We used a pragmatic and less costly CRM survey method that mainly uses qualitative methods with sub‐analysis of quantitative scoring drawn from the qualitative data, as suggested by the CRM approach. The method was used to generate data on communities' and employers' readiness scores, hence assessing their readiness for the implementation of the BFCI tailored to support a Baby Friendly Workplaces Initiatives (BFWI) for breastfeeding mothers. Qualitative key informant in‐depth interviews (KIIs) were used for in‐depth interpretation of the scores achieved and to answer the question as to why the community gave these scores. The interviews also helped to understand appropriate target for the proposed interventions. Details on the steps and procedures followed to implement the CRM survey is also described as follows:

As suggested in the CRM protocol, we followed the following four steps for the survey assessment: (i) selected six to eight people per each category for interview. These were people who knew the community and subject of interest well and who reflected the perceptions of the various stakeholders. (ii) Contacted the people identified and invited them to participate. (iii) Conducted the interviews that took 30–60 min each, which were tape‐recorded. We also took notes to complement the interview results during analysis. (iv) Scored the interview findings and determined the dimension and overall readiness scores.

The scoring was done by two trained research assistants, working independently. Any differences in scores were discussed, and when agreements were not reached, a third person independently scored the transcript to enable a majority agreed score to be generated.

The community readiness scoring was adapted from the CRM scoring guidelines (Plested et al., 2006) and contextualized for Kenya and workplace support for breastfeeding. It was done for the six dimensions and nine stages of readiness (Table 1), which served to identify key factors influencing community's preparedness to take action on BFWI issues. The dimensions assessed include

- Community efforts (programmes, activities, policies etc.): to what extent are there efforts, programmes and policies that address support for women in combining breastfeeding with work?
- Community knowledge of efforts: to what extent do community members know about local efforts and their effectiveness, and are the efforts accessible to all segments of the community?
- Leadership: to what extent are appointed leaders and influential community members supporting lactating women in combining breastfeeding with work?
- Community climate: what is the prevailing attitude of the community towards the support needed for lactating women in combining breastfeeding with work? Is it one of helplessness or one of responsibility and empowerment?
- Knowledge about the issue: to what extent do community members know about the causes of the problem and consequences, and how it impacts the local community?
- Resources for prevention efforts (time, money, people, space etc.): to what extent are local resources—people, time, money, space and so forth—available to support efforts of supporting lactating women in combining breastfeeding with work?

**TABLE 1:** Description of the nine stages of community readiness to support an intervention

Stage	Description
1. No awareness	Issue is not generally recognized by the community or leaders as a problem (or it may truly not be an issue).
2. Denial/resistance	At least some community members recognize that it is a concern, but there is little recognition that it might be occurring locally.
3. Vague awareness	Most feel that there is a local concern, but there is no immediate motivation to do anything about it.
4. Pre‐planning	There is clear recognition that something must be done, and there may even be a group addressing it. However, efforts are not focused or detailed.
5. Preparation	Active leaders begin planning in earnest. Community offers modest support of efforts.
6. Initiation	Enough information is available to justify efforts. Activities are underway.
7. Stabilization	Activities are supported by administrators or community decision makers. Staff are trained and experienced.
8. Confirmation/expansion	Efforts are in place. Community members feel comfortable using services, and they support expansions. Local data are regularly obtained.
9. High level of community ownership	Detailed and sophisticated knowledge exists about prevalence, causes and consequences. Effective evaluation guides new directions. Model is applied to other issues.

### 2.2 Study setting and period

The study was conducted in an agricultural plantation in Kericho County, Kenya. Kericho County is located in the highlands, west of the Kenyan Rift valley covering an area of 2111 km^2^ and is home to some of the largest tea companies. The study area covers about 8700 hectares of land in 112 villages with a total population of 80,000. According to administrative data during the study, the plantation had close to 16,000 employees, a third being women. A majority of the employees were casual workers whereas a minority are permanent employees working within the factories, offices and as security personnel.

The company has an organized health care system that includes one major (level 4) hospital, four (level 3) health centres and 23 (level 2) dispensaries and a comprehensive HIV/AIDs programme. Peer educators volunteer to mobilize and educate the community on health and social matters. The estate also has several social facilities including staff houses, social halls, schools and early child development centres as well as electricity and clean water supply. The study was conducted between March and June 2016.

### 2.3 Data collection

After identifying our study issue, that is, readiness to accept and practice BFWI, we defined the ‘community’ with respect to the issue. We followed the handbook for successful change community readiness assessment manual by Plested et al. (2006) to define community as ‘geographical area, a group within that area, an organisation, or any other type of identifiable “community” ’. The community of interest in this study were local leaders (village leaders, health managers and heads of administrative units), tea plantation management and knowledgeable community members.

The CRM data were collected by graduates (degrees) of public health, nutrition and other social science fields, with experience in qualitative data collection. Data collection tools were adapted from the CRM handbook (Plested et al., 2006) protocol and contextualized for Kenya and workplace support for breastfeeding. All tools were initially prepared in English and translated to Kiswahili and back translated to English to maintain consistency. A 1‐week training covering project objectives, research ethics, procedures for seeking informed consent and a comprehensive review of the data collection tools were given to all data collectors and supervisors. The training also involved practical sessions through demonstration of interviewing techniques and role‐plays as well as group discussions. The supervisors were given additional training in management of data collection, team dynamics, survey planning and logistics, observing interviews and spot checking for data quality. Piloting of the instruments was done to ensure that trainees grasp the evaluation questions and data collection tools. Debriefing sessions were held with the interviewers for sharing field experiences and to address emerging issues.

Qualitative data were collected from all the administrative blocks within the plantation. A total of 17 purposively selected men (fathers), lactating mothers, peer educators, health professionals (doctors, nurses and nutritionists), tea plantation management and other community members including grandmothers who gave consent to participate were interviewed. To ensure confidentiality, interviews were done in quiet and private areas. Qualitative interviews were tape‐recorded and transcribed verbatim to ensure accuracy.

### 2.4 Data analysis

Descriptive statistics were performed on selected socio‐demographic characteristics of participants using STATA 14 statistical software. The overall scoring process followed an easy step‐by‐step process to identify the readiness stages for each of the six dimensions of community readiness. Details on the scoring process are given in the CRM handbook (Plested et al., 2006) protocol.

Briefly, two independent professional scorers did a separate scoring in order to ensure valid results of the quantitative data.

The scoring activity started by reading through each interview in its entirety before scoring any of the dimensions in order to get a general feeling and impression from the interview. Working independently, the scorers read the anchored rating scale for the dimension being scored starting always with the first anchored rating statement. They continued to go through each dimension separately and highlighting statements that refer to the anchored rating statements. They worked stepwise until completion of reading the whole transcript of all interviews. In order to receive a score at a certain stage, a score of 1–9 was assigned based on the level of readiness reported by the interviewees.

Each scorer scored independently, and then when the independent scoring was completed, the two scorers met and discussed the scores to reach a consensus on the scores. In case of unresolved disagreement, a third person (investigator) was invited to settle the disagreement.

After scoring is completed, the average score of each dimension for each administrative unit (within the plantation) were calculated, as the sum of the average scores for each dimension divided by the number of dimensions. This was also followed by calculation of the overall stage of community readiness of the tea state company that represents all administrative units of the tea state. This was obtained by combining the sum of scores of each administrative unit within the tea state divided by the number of administrative units included in the study (*n* = 4).

To reduce the potential subjectivity bias that could potentially result by anchoring CRM surveys using pre‐defined anchored rating statements, the scoring was conducted independently by two reviewers and then combined. If discrepancies arose between reviewers, these were discussed until consensus was reached.

Qualitative data (KII) were first transcribed verbatim onto Microsoft Word and coded in NVIVO (Qsr International Pty Ltd, 2002). Themes and sub‐themes were defined for support of working mothers on breastfeeding following the CRM model. Attention was given to contradiction and diversity of experiences, perception and attitudes across the different stakeholders.

### 2.5 Ethical considerations

The study was approved by AMREF and APHRC internal Research Ethics Review Committees. A formal support communication letter was obtained from national and county health offices. Prior to undertaking interviews, verbal consent was obtained from all adults to help open and free discussion on issues after explaining the situation and reaching an agreement with all participants.

In the present reporting, we provide analysis of quantitative CRM surveys as well as qualitative findings at institutional (company) level but not individual community units or respondents to ensure confidentiality. Furthermore, as the study findings will be used to design interventions at company level, we preferred to report institutional level analysis findings.

## 3 RESULTS

### 3.1 Socio‐demographic characteristics of study participants

Table 2 presents socio‐demographic characteristics of community leaders and other community members. From a total of 17 participants, the majority (52.9%) were male and belong to Kalenjin ethnic group. Most of the participant were married (82.3%) and under 40 years of age (70.6%).

**TABLE 2:** Socio‐demographic characteristics of community leaders and other community members (*n* = 17)

Characteristics	Number	Per cent
Sex		
Male	9	52.9%
Female	8	47.1%
Age (years)		
20–29	6	35.3%
30–39	6	35.3%
40–49	5	29.4%
Ethnic group		
Kalenjin	9	52.9
Kisi	2	11.8
Luhya	1	5.9
Kipsigis	5	29.4
Educational status		
Primary school	2	11.8
Secondary school	4	23.5
High school completed	6	35.3
More than high school	5	29.4
Marital status		
Single	3	17.6
Married	14	82.3
Role in the community/company		
Manager	2	11.8
Supervisor/team leader	3	17.6
Admin (security)	1	5.9
Pear educator	1	5.9
Casual worker (tea pluck)	3	17.6
Health worker	2	118
Village leader	4	23.5
Total	17	100

### 3.2 Community readiness scores

The mean (SD) readiness score was 7.3 (1.9) (Table 3). This corresponds to the third highest level of the nine stages of the community readiness or the ‘stabilization stage’.

**TABLE 3:** Community readiness scores obtained from a sample of community leaders and other community members (*n* = 17)

Dimensions	Community efforts	Knowledge of community efforts	Leadership	Community climate	Knowledge of the issue	Resources
Mean score (SD)	7.5 (2.1)	6.6 (2.3)	8.3 (1.9)	7.3 (1.6)	7.3 (1.6)	6.6 (1.9)
Overall readiness stage (SD)	7.3 (1.9)	(Stabilization stage: Activities are supported by community leaders and other community members)				

**FIGURE 1:**
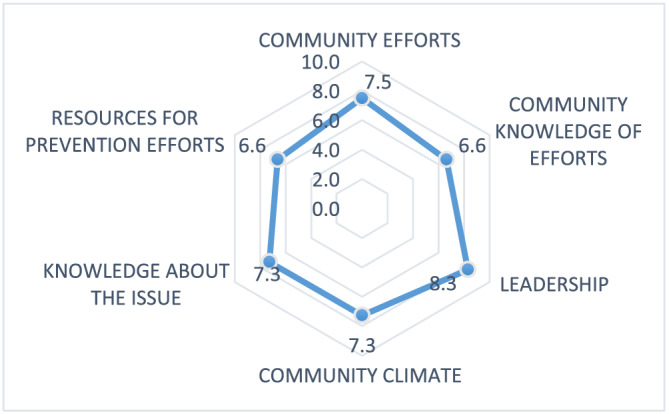
Community readiness assessment graph showing the average score per dimension for participants from four administrative units

Figure 1 interprets scores within each dimension showing the mean readiness score for leadership as the highest of all the dimensions (8.3 ± 1.9) followed by community efforts (7.5 ± 2.1). The lowest score was seen for knowledge of efforts (6.6 ± 2.3) and availability of resources (6.6 ± 1.9).

Results related to community efforts suggest that programmes and policies are in place, addressing support for combining breastfeeding with work by local decision makers, company managers/supervisors and other community members. However, more efforts are still needed so that community members feel comfortable using services, and local data are regularly obtained. In addition, actions towards detailed and sophisticated knowledge about prevalence, causes and consequences of unsupported working mothers to combine their work with breastfeeding are needed.

### 3.3 Community efforts

The mean (±SD) score for community efforts was 7.5 (2.1) out of the nine maximum achievable. This was following the responses from managers and supervisors in the plantation about the existing community efforts scores: community issues (i.e., social, health and safety issues), resources and the company's role in helping working mothers combine breastfeeding with work. The high score was also consistent with responses of participants which they mainly attributed to the existing system or the company's policy as well as government structures supporting employed mothers to combine breastfeeding with work are in place, as evidenced by some of their responses:

…. The first policy is that the women should be given time to breastfeed that child and also be paid, also she should be given light jobs until she is done with breastfeeding …. (Plantation Supervisor)

This shows the fact that the company has enacted policies that promote working mothers to have time to breastfeed and do lighter work that lowers the burden of lactating mothers. This was also supported by community leaders, the following quote elucidating the same.

… because working mothers are given 3 months paid leave to take care of the baby first … and after she comes back, again they give her chance to go to work, come back and take care of the baby and go back to work again …. (Village Elder)

The high readiness score also revealed the presence of responsive leadership and supportive work arrangements within the company to help working mothers.

… when people are at work we employ female security to be in the villages, as the mothers are at work … so, during day time we assign female security … so that they can take care of the kids …. (Plantation Manager)

Furthermore, the presence of strong support from local government and the ministry of health and trained and experienced staffs is evident.

…. Our County Health Committee (CEC) member has been informing the community about exclusive breastfeeding via the radio, the benefits of breastfeeding, the need to breastfeed the babies and that information. A lot of people were concerned, both men and women, in terms of breastfeeding and I believe when we go out there to the community and bring in the idea, they already know the benefits of breastfeeding, they will buy‐in to what we want to do …. (Female, 47 years, Doctor [CHMT])

According to this health worker from the Ministry of Health, the government is also supporting the initiative through media advocacies and other information communication channels.

### 3.4 Community's knowledge of efforts

A modest score of 6.6 (± 2.3) out of nine achievable was recorded for preparedness from community's knowledge of efforts. Although there is enough information to justify efforts and activities are underway, practical support from administrators or community decision makers was lacking or not adequate.

… for now, workers have been given breastfeeding guideline from the company, which I have … but there are those who will be affected especially those employees who are called term‐contract employees or temporary employees, who work for two months then stop and go …. So you will find that such a person will have to first write and say that … to write a letter requesting that she has a child and request to be given guideline or to be given break to go and check on the child. (Village Elder)

This shows that unlike the knowledge and motivation, some bureaucratic arrangements and practical administrative arrangements are still causing challenges.

From the employer's (the company) perspective, the knowledge of efforts are better understood, but they also acknowledge some gaps which, to the employer, are attributable to the large number of employees and relatively longer (annual) time taken to review policies and guidelines.

They know normally what happens actually it is them who request like the policy, we normally review on a yearly basis, it goes to the field, they discuss amongst themselves they give comments and then a committee sits, we normally review on a yearly basis and after approval it goes to the employees. (Plantation Manager)

### 3.5 Leadership and community climate

Leadership (i.e., the extent to which appointed leaders and influential community members are supportive of the issue) and community climate (i.e., the prevailing attitude of the community towards the issue cores was high, 8.3 [±1.9] and 7.3 [±1.6], respectively: one of the highest scores of all the dimensions). These results suggest, in accordance with the CRM handbook, that (1) community members feel comfortable using services, and they support expansions, mainly due to strong support and acceptance as well as engagement of the leadership, and (2) the prevailing attitude of the community towards the issue is one of responsibility and empowerment. The following themes from the KIIs were used to interpret the leadership and community climate scores: presence of forums and community engagement participation events, like community meets (barazas) helped to raise the level of freedom and high level of engagement:

… when forums are called for, that is when they usually discuss those issues. If there are Baraza's, that is when they are encouraged. That is how they are involved. But mostly, it is at the workplace, yes, at work, one is given freedom. In case of any problem you have, raise it … especially because we are at work throughout, Sunday is a church day, and mostly they are told it is during work time that if someone has a problem he/she should say it so that he/she can be assisted. (A village leader)

In the same way, fathers, peer educators and team leaders affirmed the support and commitment of employer and leaders at all levels of the company structure. They were reported to be fully committed and willing to support breastfeeding mothers at workplace that enhanced mothers' experience of combining breastfeeding with work.

… when we meet in forums, many staff tell us that lactating mothers should be assigned light work and work whereby she could leave early, especially the one where people leave work at one o'clock …. Company managers encourage that lactating mothers should be given the chance to leave early at ten o'clock to go and check on the baby …. So, the management has set it that one is free to ask for the work where they leave early so that she can take care of the baby …. (Village Elder)

This was also supported by responses from onsite job supervisors:

…. On my side, the management has given us the opportunity that if there is a lactating woman having a problem in breastfeeding, I will take it upon myself to give her the time to tell me her problems then I give her the support she requires …. (Plantation Supervisor)

The community's attitude of responsibility and empowerment is also reflected in many ways. The presence of community‐initiated criticism of reluctant mothers who are not breastfeeding babies because they are worried about earning money is one way that strong ownership was voiced:

… I give a case …, there are some mothers who are running after money until they forget to go and attend to the children … they normally speak on her behalf … they ask the team leader to give her permission and go to breastfeed … there are those who say this woman has a young child who needs to be taken care of, why is she running after money issues until she is about to forget …. (Village Elder)

The presence of ongoing discussions about the issue at all levels was a good example of knowledge of the issue in the community. The discussions do raise some issues regarding why women are not able to practice exclusive breastfeeding in this community as well:

… I think the community would really love to support the mothers to breastfeed their babies … and one, it's just that everybody is looking for resources and that is what has made the mothers again not breastfeed …. (KII, a doctor, member of the Community Health Management Team [CHMT])

### 3.6 Knowledge of the issue of combining breastfeeding with work

The readiness score for knowledge of the issue (i.e., the extent to which community members know about the causes and consequences of the issue) was also among the highest in the dimensions [7.3 ± 1.6]. This result suggests, in accordance with the CRM handbook (Plested et al., 2006), that a larger proportion of people in the community have adequate knowledge of the level and types of support needed for lactating mothers who are working. Many of the leaders from the employers' side, starting from site supervisors to key top managers, believe that productivity of women will increase as they combine work with breastfeeding rather than being worried about breastfeeding when working.

… I will 100 percent, the productivity of women will increase, if we help them … and all leaders will not be worried when seeking working mothers … imagine what may happen if they left the child and come for work …, we will be comfortable so our productivity will definitely improve. (Plantation Manager)

Community and leaders' knowledge of the benefit of breastfeeding and understanding of the potential challenges of breastfeeding was reported to be the key to the success of the support given:

… I think everybody knows the benefit of breastfeeding and the challenge in terms of breastfeeding … everyone knows that the women need to be considered and the babies, we are not only looking at the breastfeeding mothers but we are looking at the baby who is being breastfed. So, I believe all of us think that we really need to give consideration to the breastfeeding mothers …. (CHMT)

This shows that the CHMT, as a leader, understands the challenges and promises to take actions in the future. This is believed to be critical to the success of support needed to working mothers as they are the one who make decisions on time, money and space/facilities.

### 3.7 Resources

The readiness score for resources (i.e., the extent to which people, time, money and space are available to support efforts), 6.6 + 1.9, was good but was the lowest compared to other dimensions. This suggests that the community has enough information available to justify efforts and some activities are underway but are not fully supported by administrators or community decision makers, or staff are not well trained and experienced (Plested et al., 2006). The following themes from the KIIs were used to interpret the resources scores: people, time, money, space/facilities and religious writings. Supervisors and line managers as well as elders are happy with the existing health information dissemination activities but wished to have a better way, like presence of a home‐based counsellor to support their activities:

…. On my side, I would recommend the way the company is bringing people to teach mothers … it should continue this way, because this information is good and helps people like now you have asked me. …, but if we can get someone going door to door a counsellor that will be good because you will not get so many questions …. (Plantation Supervisor)

The plantation supervisor identified a gap in the system that actually does exist as identified by a peer educator in the company who was interviewed and mentioned the presence of such support systems. This highlights a potential problem with information flowing through all levels of the system about the support systems available:

We teach them in the morning, in the main master, if we have village assemblies we also teach, even the company is doing something good, as peer educators, we are going to a certain village to teach a particular thing, the management helps us to go and teach, they don't discourage us even if we are outside the issues of the company …. (Peer Educator)

… I think the information is available especially to pregnant women and breastfeeding mothers. But, as I mentioned earlier, the social support that the women get is more than just the mother and the baby. We have the mother‐in‐law or maybe the grandmother of the child, both from the mother and the father … they are able to be supported both from their work place and the home to be able to breastfeed. (CHMT)

## 4 DISCUSSION

Today, returning to work while still breastfeeding presents a challenge in many parts of the world (Chuang et al., 2010; Neilsen, 2004; Thomas‐Jackson et al., 2016). Maternal return to work, especially within 6 months after giving birth (Neilsen, 2004), is a major barrier to the initiation and continuation of breastfeeding (Thomas‐Jackson et al., 2016). The lack of breastfeeding breaks and resources to promote breastfeeding, lack of support from employers and colleagues and inadequate facilities are among the challenges faced by employed mothers who want to continue breastfeeding (Bonet et al., 2013; Chuang et al., 2007; Hawkins et al., 2007). Early cessation of breastfeeding may also cause disappointment and distress to the mothers and health problems for themselves and their infants (WHO/UNICEF, 2015).

As such, support to lactating mothers in different forms like giving reassurance, praise, information and an opportunity to discuss problems and ask questions and others is essential. Conducting a community readiness assessment by dimension about the level of such support and the need for further developments to support interventions targeted at breastfeeding mothers is the key to design tailored interventions. In this study, we generated evidence on the level of preparedness and readiness of local leaders, employers and community members in an agricultural plantation in rural Kenya to support employed mothers, who are also lactating, combine breastfeeding and work.

Our findings show that the overall readiness score of the community and local leaders was 7.3 out of nine stages, corresponding to the state of stabilization. According to Community Readiness Handbook (Plested et al., 2006), communities scored to this phase usually exhibit a wider acceptance of the activity with potential availability of trained and experienced staff in the area of interest. Likewise, our results also were consistent with the ratings of the handbook showing that support given to lactating mothers in combining breastfeeding with work in the area is highly welcomed by employers, local authorities and the community at large.

Dimensional analysis also showed that a highest score of readiness was observed by the leadership showing a promising step to ensure the sustainability of additional interventions. This is consistent with findings of multiple studies that demonstrated improved breastfeeding outcomes when leaders adopt the steps (Britton et al., 2007; Dodgson et al., 2004; Johnston & Esposito, 2007). This is because management can encourage supervisors to work with breastfeeding employees in making reasonable accommodations to help them reach their breastfeeding goals and can encourage other employees to exhibit a positive, accepting attitude (Tsai, 2013). Furthermore, with the existing (inadequate) level of resources and community's knowledge of efforts, a high level of preparedness from leaders to support breastfeeding and working mothers makes things easier, so that community members feel comfortable using services and support expansions (Tsai, 2013).

A relatively low level of the community's knowledge of efforts and resources implies that future proposed interventions in such settings should be geared particularly towards increasing knowledge and availing basic resources to ensure sustainability, a higher level of readiness and continued support.

Findings from our study also highlight that the community is highly likely to support working mothers to combine breastfeeding with work, thus supporting international legislation to protect the rights of women breastfeeding in the workplace (WHO/UNICEF, 2015).

Returning to work while still breastfeeding presents a challenge in many parts of the world (Chuang et al., 2010; Neilsen, 2004; Thomas‐Jackson et al., 2016), but in this study, we found out a high level of readiness and preparedness among local leaders, employers and community's efforts to follow leaders in supporting working mothers to combine breastfeeding with work. As such, employers, programmers and policy makers could consider a breastfeeding‐friendly workplace intervention, through the provision of lactation rooms and breast pumping breaks for working and lactating mothers to express breast milk for children and/or allowing flexi hours as a critical element, which may increase a mother's intention to continue breastfeeding after returning to work.

Availability of resources like adequate facilities for pumping and storing milk and community's knowledge of efforts that promote breastfeeding were critical determinants and shortcoming of readiness for support in the studied population. Similar studies in Kenya (Lakati et al., 2002a) and globally (Noble & ALSPAC Study Team. Avon Longitudinal Study of Pregnancy and Childhood, 2001; Tsai, 2013) have also indicated that availability of resources, organizational support for breast pumping breaks and encouragement from colleagues and supervisors to use breast pumping are significant predictors of continued breastfeeding for more than 6 months after returning to work.

Plested et al. (2006), in their Community Readiness Handbook, state that for an intervention to be effective, each dimension should be at an equal stage of readiness. Therefore, based on our results, an initial focus on preparation for an intervention to improve breastfeeding practices for mothers returning to work should be on increasing knowledge of efforts and resources. The study has highlighted that the first starting point for an intervention in this setting will be to introduce innovative approaches to increase knowledge.

The findings of this study should be interpreted cautiously for the following limitations. Though views and opinions of local leaders, lactating mothers and key community groups are included, views of pregnant mothers on preparedness of the community and employers were not included. This could be a potential reason for missing some real perspectives of mothers. This was not possible because the CRM focuses on the level of preparedness from the community's perspective because this is where the intervention will be delivered from to support lactating mothers.

The observed level of preparedness among employers and community members in rural Kenya may not necessarily be reflective of other employers in Kenya or elsewhere. This is mainly related to the fact that working conditions and organizations cultures as well as values are not necessarily similar across employees and different companies. Furthermore, as this study collected data from a small sample of selected business units within a large tea estate farm, the CRM scores may not be representative of the Kericho county and the area as a whole. But it should also be noted that in addition to the community within the plantation being relatively homogenous, we also collected data from a wide stakeholders' perspective, which is highly likely to reflect views of all managers and communities within the tea estate and to the local area. The data are certainly sufficient for planning the next steps for intervention in this tea estate.

A strength of the study was to use the CRM to assess community readiness to help to plan the next steps for intervention. To the best of our knowledge, this is the first study assessing communities' readiness for change in maternal and child health interventions, particularly support needed for breastfeeding.

## 5 CONCLUSION

Generally, the study highlighted the existence of a high level of preparedness and readiness for support for breastfeeding in mothers returning to work in the agricultural plantation that employs a large number of women. Local leaders and managers from top level to onsite supervisors have showed strong interest and preparedness to support working and lactating mothers in very many ways extending from arranging flexi hours or breastfeeding breaks to amendment of organizational policies. However, knowledge of efforts and availability of resources remained suboptimal. Future interventions should focus on ways that enable community members to feel comfortable using services and support expansions. They should also help towards creating detailed and sophisticated knowledge about prevalence, causes and consequences of breastfeeding and working for mothers.

## CONFLICTS OF INTEREST

The authors declare that they do not have any competing interests.

## CONTRIBUTIONS

PG, EKM and LK designed the study. TAZ and EA developed analyses parameters, developed objectives and secured support. TAZ and EA conducted detailed analyses and were involved in the write up and synthesis of the findings. All authors read and approved the final manuscript.

### DATA AVAILABILITY STATEMENT

The data that support the findings of this study are available from the corresponding author upon reasonable request at tadalzerfu@gmail.com.
